# Antimicrobial Resistance and Phylo-Groups of *Escherichia coli* at the Human–Primate Interface in Gabon: A One Health Study

**DOI:** 10.3390/antibiotics15050446

**Published:** 2026-04-29

**Authors:** Marie-louise Mawili Mounguengui, Richard Onanga, Anicet-Clotaire Dikoumba, Yann Mouanga-Ndzime, Gabriel Falque, Aicha Mohamed Ali, Léonce F. Ondjiangui, Leresche E. D. Oyaba Yinda, Ivan Mfouo-Tynga, Linaa Y. Okomo Nguema, Jean Nzue Nguema, Thierry A. G. Tsoumbou, Serge E. Dibakou, Désiré Otsaghe Ekore, Barthélémy Ngoubangoye, Sylvain Godreuil

**Affiliations:** 1 Bacteriology Laboratory, Franceville Interdisciplinary Medical Research Center, Franceville P.O. Box 769, Gabon; dikoumba@hotmail.com (A.-C.D.); yann_mouangandzime@yahoo.fr (Y.M.-N.); leoncefauster@gmail.com (L.F.O.); okomoyasmine@gmail.com (L.Y.O.N.); otsaghe16@gmail.com (D.O.E.); genistha@hotmail.com (B.N.); 2 Viral Disease Emergencies Unit, Franceville Interdisciplinary Medical Research Center, Franceville P.O. Box 769, Gabon; gabfalque@hotmail.fr; 3 Peltier General Hospital, Djibouti 2073, Djibouti; aichamedali30@gmail.com; 4 Faculty of Medicine and Health Sciences, Masuku University of Science and Technology, Franceville P.O. Box 901, Gabon; oyabaeven@gmail.com; 5 Retroviral Infections and Associated Diseases Unit, Franceville Interdisciplinary Medical Research Center (CIRMF), Franceville P.O. Box 769, Gabon; tivansdavids2012@gmail.com; 6 Primatology Center, Franceville Interdisciplinary Medical Research Center, Franceville P.O. Box 769, Gabon; nguemanzue14@gmail.com (J.N.N.); thierrytsoumbou@yahoo.fr (T.A.G.T.); sergeely@live.fr (S.E.D.); 7 Hematology Department, Saint-Éloi University Hospital, University of Montpellier, 34090 Montpellier, France; s-godreuil@chu-montpellier.fr; 8 Microbiology Department, Lapeyronie University Hospital, University of Montpellier, 34295 Montpellier, France

**Keywords:** *Escherichia coli*, antibiotic, antimicrobial resistance, phylo-groups, non-human primates

## Abstract

Background/Objectives: Antimicrobial resistance (AMR) is a global threat to human, animal, and environmental health. Among bacteria, *E. coli* is frequently used as a key indicator of AMR. Despite their genetic proximity to humans, studies on AMR in Non-Human Primates (NHPs) remain limited, particularly in semi-anthropized environments. This study aims to characterize the antibiotic resistance profiles and phylo-groups of *E. coli* isolated from NHPs and humans at a primatology center. Methods: A total of 143 stool samples were collected, including 125 from NHPs and 18 from humans. Isolates were cultured on Eosin Methylene Blue agar and then identified by MALDI-TOF mass spectrometry. Antibiotic susceptibility was assessed using the Kirby–Bauer disk diffusion method, with 30 antibiotics following CASFM-EUCAST recommendations. *E. coli* phylo-groups were characterized by quadruplex PCR according to the Clermont method, targeting the genes. Results: A total of 122 *E. coli* isolates (85.31%) were recovered, with comparable prevalence observed across NHPs and human staff. More than half of the isolates (55.74%) were resistant to at least one antibiotic tested, and 12.3% were classified as multi-drug resistant (MDR). Resistance rates of isolates in *Mandrillus sphinx*, *Pan troglodytes*, and humans were 50.6%, 57.7%, and 80.0%, respectively, with no significant statistical difference (*p* = 0.11). A single Extended-Spectrum Beta-Lactamase (ESBL) producing isolate was identified in the mandrill. Phylo-group analysis revealed the dominance of group A (50%), followed by groups B1, D, and C. Conclusions: Resistance profiles and phylo-group distribution among NHPs could suggest bacterial exchange and potential for cross-transmission of AMR within the shared environment.

## 1. Introduction

Antimicrobial resistance is a global threat with significant consequences for human, animal, and environmental health [1]. *Escherichia coli* (*E. coli*), a commensal bacterium ubiquitous in many ecosystems, including the gastrointestinal tract of humans and animals, is often employed as a key indicator of AMR [2]. This species readily crosses interspecies barriers, facilitated by its rapid reproductive capacity and mechanisms such as horizontal gene transfer, conjugation, and mutation [3,4,5]. Given its widespread involvement in various health processes, the presence of AMR in *E. coli* constitutes a major challenge for both human and animal health [4].

Assessing AMR in Non-Human Primates (NHPs) is crucial due to their close genetic proximity to humans and their frequent interactions with semi-anthropized environments, which collectively promote interspecies transmission [6]. While the majority of research concerning resistant commensal bacteria has focused on farm animals, studies focusing on NHPs remain limited [7]. Nevertheless, existing literature has demonstrated that humans and primates share similar strains of resistant bacteria, highlighting the bidirectional nature of transmission [8,9].

Further research conducted in Kenya on captive baboons at the Primate Research Institute, and in Uganda on wild primates near two national parks, has identified resistance to common antibiotics, such as ampicillin, sulfamethoxazole, and tetracycline [7,10]. Similarly, in Gambia, a study by Foster-Nyarko et al. (2020) revealed that some antibiotic-resistant *E. coli* isolates were closely related to those found in humans, suggesting potential interspecies transmission of resistance genes [11]. Locally, research conducted in Gabon on western lowland gorillas in Moukalaba-Doudou National Park (MDNP) demonstrated a high level of resistance to the beta-lactam family, followed by resistance to fluoroquinolones and low resistance to aminoglycosides [12].

In Gabon, NHPs housed in the semi-captive environment of the CIRMF (Interdisciplinary Medical Research Center of Franceville) Primatology Centre maintain regular and close contact with human staff.

This proximity creates many opportunities for the bidirectional transmission of microorganisms, including antibiotic-resistant bacteria. Previous studies at this site have documented the exchange of *Staphylococcus aureus* strains between NHPs and caretakers, which has occasionally led to severe clinical outcomes [13], and human caretakers, which has occasionally resulted in severe infections and even the death of a gorilla [14]. However, the extent of cross-species exchange regarding *E*. *coli,* a key indicator of AMR dissemination, remains poorly understood. *E. coli* phylo-groups, particularly B2 and D, are frequently associated with extraintestinal infections, thereby underscoring the public health risks associated with resistant isolates from NHPs. Identifying phylo-groups in primates and comparing them with *E. coli* isolates from human staff would provide a crucial understanding of the dynamics of resistance and interspecies transmission within this high-contact setting [11,12,15,16].

Unlike isolated sylvatic (forest) environments, this center is characterized by frequent, high-intensity interactions between humans and NHPs during daily husbandry, clinical care, and enclosure maintenance. This sustained proximity creates a viable pathway for the bidirectional transfer of resistant bacteria. Furthermore, the administration of antibiotics to NHPs at this facility, a factor rarely present in wildlife studies, provides a unique opportunity to evaluate antimicrobial selection pressure in a controlled setting. Collectively, these factors establish the center as a critical longitudinal observatory for understanding the dynamics of AMR in Gabon.

Therefore, this study aimed to characterize the AMR profiles and phylo-groups of *E. coli* isolated from *Pan troglodytes*, *Mandrillus sphinx*, and human staff at the CIRMF Primatology Centre in Gabon. Specifically, by comparing resistance patterns and phylo-group distributions among these three populations that share a unique ecological setting, we aimed to identify potential factors contributing to the observed similarities between human staff and NPH isolates. This work provides novel insights into the dynamics of AMR in shared environments and reinforces the importance of a One Health approach in monitoring and preventing bacterial transmission across species.

## 2. Results

### 2.1. Demographic Data and Human–Animal Interactions

A total of 143 samples were collected from 18 human staff members (56% male, 10/18), 95 mandrills (*Mandrillus sphinx*) (54% male, 51/95), and 30 chimpanzees (*Pan troglodytes*) (67% male, 20/30). While mandrills lived in semi-captive conditions, chimpanzees were maintained in strict captivity. Frequent contact between humans and NHPs occurs through daily activities, including enclosure maintenance, feeding, and medical care.

### 2.2. Distribution of E. coli Isolates

Initial identification of the 143 isolates based on colony morphology on EMB agar was followed by MALDI-TOF MS confirmation. A total of 122 isolates (85.3%) were confirmed as *E. coli*, while 21 (14.7%) were identified as other species. The isolation rates were comparable across the study populations. An overall rate of 86.7% (107/125) was observed and broken down by species: *Mandrillus sphinx* 85.3% (81/95), *Pan troglodytes* 86.7% (26/30), and human staff 83.3% (15/18).

### 2.3. Antibiotic Resistance Profiles

**Figure 1:**
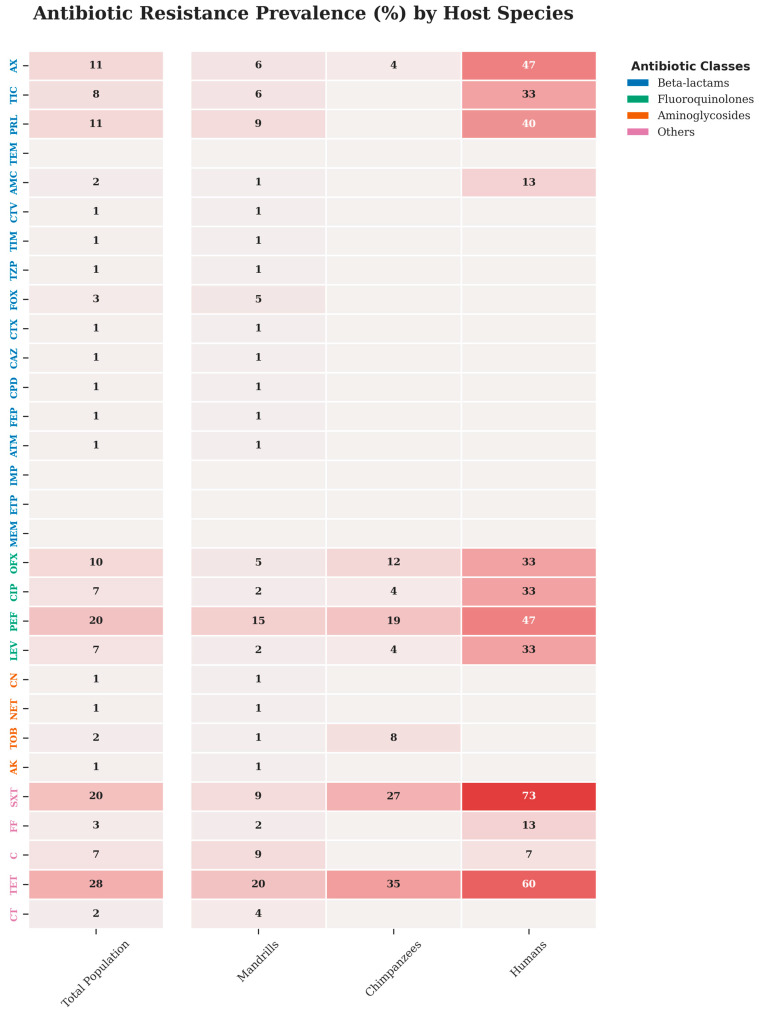
Comparative landscape of antimicrobial resistance prevalence across host species. Heatmap depicting the frequency of phenotypic resistance among *E. coli* isolates for each tested antimicrobial agent. (**Right**) Resistance profiles stratified by host origin: Humans, Chimpanzees (*Pan troglodytes*), and Mandrills (*Mandrillus sphinx*). (**Left**) Aggregate antimicrobial resistance prevalence for the entire study population (Total Population). Numerical values represent the percentage of resistant isolates per category; null values are intentionally omitted to highlight significant resistance clusters. The color gradient reflects resistance intensity. Antimicrobial labels on the y-axis are color-coded by therapeutic class: β-lactams (blue), fluoroquinolones (green), aminoglycosides (orange), and other classes (magenta) Legend: AX: amoxicillin, AMC: amoxicillin/clavulanic acid, TIC: ticarcillin, TIM: ticarcillin/clavulanic acid, PRL: piperacillin, TZP: piperacillin/tazobactam, CTV: ceftazidime-avibactam, FOX: cefoxitin, CTX: cefotaxime, CPD: cefpodoxime, CAZ: ceftazidime, FEP: cefepime, ATM: aztreonam, IMP: imipenem, ETP: ertapenem, MEM: meropenem, TEM: temocillin, AK: amikacin, CN: gentamicin, NET: netilmicin, TOB: tobramycin, OFX: ofloxacin, LEV: levofloxacin, CIP: ciprofloxacin, PEF: pefloxacin, SXT: trimethoprim-sulfamethoxazole, TET: tetracycline, FF: fosfomycin, C: chloramphenicol; CT: colistin.

Across the study population, 55.7% (68/122) of *E. coli* isolates were resistant to at least one of the 30 antibiotics tested, and 12.3% (15/122) were MDR. The highest resistance rates were observed for tetracycline 27.9%, trimethoprim-sulfamethoxazole 20.5%, and pefloxacin 19.7%. No resistance was detected against carbapenems (imipenem, ertapenem, meropenem). Overall resistance prevalence in *Mandrillus sphinx*, *Pan troglodytes* and humans were 50.6% (41/81), 57.7% (15/26), and 80.0% (12/15), respectively, with no significant difference (*p* = 0.11). *Mandrillus sphinx* and *Pan troglodytes* showed the rate of resistance to tetracycline with 19.7% (16/81) and 34.6% (9/26), respectively. In human staff, the rate of resistance to tetracycline was 60% (9/15), and the highest resistance was observed to trimethoprim-sulfamethoxazole with 73.3% (11/15). A single ESBL-producing isolate was identified in a mandrill (Figure 1).

### 2.4. Distribution of Phylo-Groups

Phylo-group diversity was observed in 94.3% (115/122) of the isolates. The phylo-group distribution was characterized by the dominance of phylogroup A, 50% (61/122) representing 51.8% (42/81) in *Mandrillus sphinx*, 57.6% (15/26) in *Pan troglodytes*, although it was less frequent in humans, with a rate of 26.7% (4/15).

**Figure 2:**
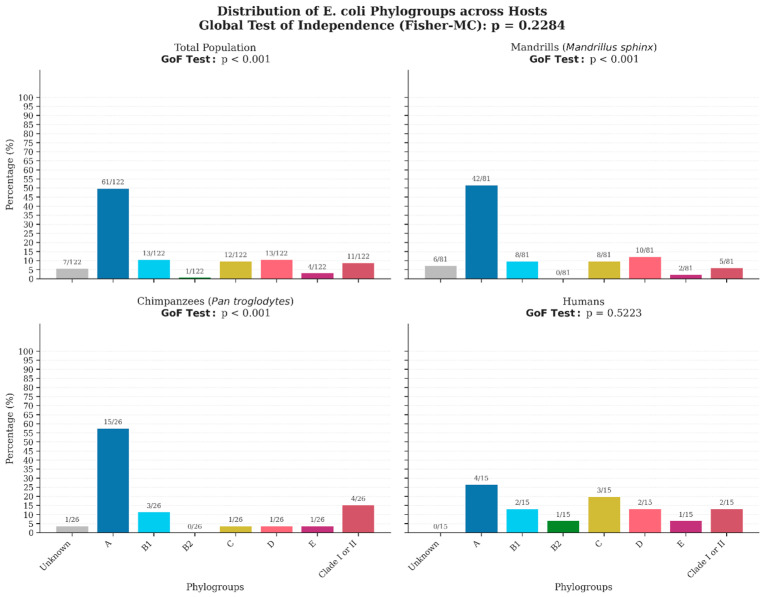
Distribution and prevalence of *E. coli* phylo-groups across human staff and non-human primate hosts. Bar charts display the relative abundance (%) of *E. coli* phylo-groups (A, B1, B2, C, D, E, Clade I/II, and Unknown) identified in the study population. Panels are stratified by host species: Mandrills (*Mandrillus sphinx*, n = 81), Chimpanzees (*Pan troglodytes*, n = 26), and Humans (n = 15), alongside the total population (n = 122). Ratios above bars indicate the absolute number of isolates observed relative to the total sample size for each group. Statistical significance was assessed using Monte Carlo simulations (105 iterations) to account for small sample sizes in specific categories. The global *p*-value (Fisher-MC, top title) corresponds to a test of independence between host species and phylogroup profiles. Individual panel *p*-values represent Chi-square goodness-of-fit (GoF) tests evaluating deviation from a uniform distribution null hypothesis.

Phylo-group B1 accounted for 9.9% (8/81) in *Mandrillus sphinx*, 11.5% (3/26) in *Pan troglodytes*, and 13.3% (2/15) in humans. Phylo-groups C and D were detected in *Mandrillus sphinx* at 9.9% (8/81) and 12.3% (10/81), respectively, and at lower rates in *Pan troglodytes*, at 3.8% (1/26) each. In human staff, phylogroups C (20.0% (3/15) and D (13.3% (2/15) were relatively common, while phylogroup B2 was only identified in human staff 6.7% (1/15). Seven isolates (5.7%) remained unclassified by the Clermont method (Table S1). Detailed comparative prevalence rates are provided in Figure 2.

### 2.5. Phylo-Groups Associated with Antibiotic Resistance

**Figure 3:**
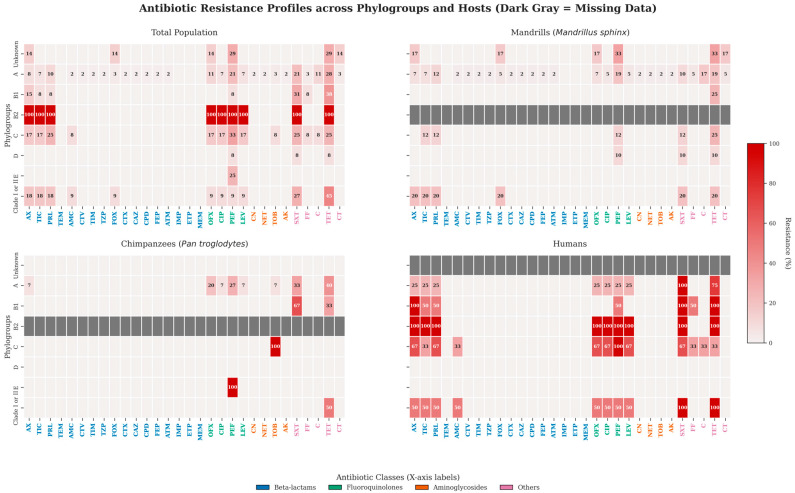
Antibiotic resistance profiles of *E. coli* phylogroups across human staff and non-human primate hosts. Heatmaps illustrate the prevalence of antibiotic resistance (%) among *E. coli* isolates, stratified by host species (Mandrills [*Mandrillus sphinx*], Chimpanzees [*Pan troglodytes*], and Humans) alongside the total population. The y-axis represents the identified *E. coli* phylogroups. Resistance prevalence was calculated as the proportion of resistant isolates relative to the total number of isolates tested for each specific antibiotic; missing data were strictly excluded from the denominators, and intermediate phenotypes were classified as susceptible. The color gradient, ranging from white (0% resistance) to dark red (100% resistance), indicates the resistance rate, with exact non-zero percentage values annotated within the cells (zeros are omitted for visual clarity). Dark gray cells containing a dash (“-”) denote phylogroup-antibiotic combinations for which no isolates or testing data were available. To highlight resistance patterns across distinct pharmacological mechanisms, antibiotic abbreviations on the x-axis are color-coded by class: Beta-lactams (blue), fluoroquinolones (green), aminoglycosides (orange), and others (purple). Legend: AX: amoxicillin, AMC: amoxicillin/clavulanic acid, TIC: ticarcillin, TIM: ticarcil-lin/clavulanic acid, PRL: piperacillin, TZP: piperacillin/tazobactam, CTV: ceftazidime-avibactam, FOX: cefoxitin, CTX: cefotaxime, CPD: cefpodoxime, CAZ: ceftazidime, FEP: cefepime, ATM: az-treonam, IMP: imipenem, ETP: ertapenem, MEM: meropenem, TEM: temocillin, AK: amikacin, CN: gentamicin, NET: netilmicin, TOB: tobramycin, OFX: ofloxacin, LEV: levofloxacin, CIP: ciprofloxacin, PEF: pefloxacin, SXT: trimethoprim-sulfamethoxazole, TET: tetracycline, FF: fosfomycin, C: chloramphenicol; CT: colistin.

Overall, more than half of the *E. coli* isolates belonging to phylo-groups A, B1, C and I/II showed resistance to at least one antibiotic. Resistance rates were 57.4% (35/61) in phylo-group A and 63.6% (7/11) in phylo-group I/II. Phylo-group D was characterized by a lower prevalence of resistance, at 25.0% (3/12). Although phylo-group B2 showed a resistance rate of 100%, the small sample size limits interpretation (Figure 3).

### 2.6. Multivariate Analysis of Resistance Profiles

**Figure 4:**
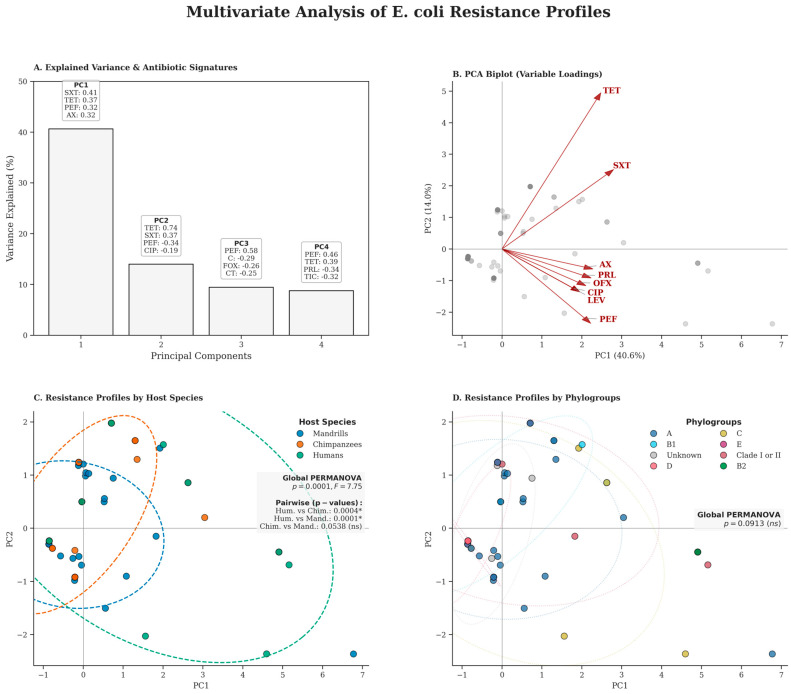
Multivariate analysis of *E. coli* antibiotic resistance profiles across host species and phylogenetic groups. (**A**) Variance distribution and antibiotic signatures. The bar plot illustrates the percentage of total variance captured by the first four principal components (PC1–PC4), cumulatively explaining 72.8% of the data variability. Annotated boxes highlight the top four antibiotic contributors to each axis, with values indicating their respective loadings. PC1 (40.6%) primarily reflects a multidrug resistance (MDR) gradient, while PC3 isolates specific quinolone resistance signatures (e.g., PEF). (**B**) PCA Biplot of variable loadings. Individual isolate projections (light gray) are superimposed with loading vectors (red arrows) for the eight most influential antibiotics. The orientation and length of the vectors indicate the strength of the correlation between specific antibiotics and the principal components. (**C**) Host-driven structure of resistance profiles. Scatter plot of the first two principal components color-coded by host species. Ellipses represent the 95% confidence intervals for Humans (green), Chimpanzees (orange), and Mandrills (blue). Statistical significance was assessed using a global PERMANOVA (9999 permutations) followed by pairwise comparisons with Bonferroni correction (significance threshold: *p* < 0.0167). The results indicate that the host environment is the primary driver of resistance profiles, with a marked separation between human staff and non-human primate populations. *, significant; ns, not significant. (**D**) Phylogenetic structure of resistance profiles. Scatter plot color-coded by *E. coli* phylogroups. In contrast to host species, phylogenetic background did not significantly structure the resistome (Global PERMANOVA, *p* = 0.0913), suggesting that environmental selective pressures and horizontal gene transfer override vertical inheritance in shaping resistance patterns. All analyses were performed on ordinal-encoded resistance data (susceptible = 0, intermediate = 1, and resistant = 2) derived from 30 tested antibiotics.

A multivariate approach to the antibiotic resistance profiles of *E. coli* in different host species and phylogenetic groups was performed using principal component analysis (PCA). The PCA reveals that the first four principal components (PCs) explain 72.8% of the total variance, ensuring excellent representativeness of the data structure. PC1 was primarily defined by resistance to trimethoprim-sulfamethoxazole (SXT) and tetracycline (TET), which acted as the main drivers of isolate dispersion. PC2 was further influenced by fluoroquinolones (PEF and CIP). The overall PERMANOVA analysis revealed significant differences in antimicrobial resistance profiles between host species (*p* = 0.0001, F = 7.75). Pairwise comparisons showed that while humans differed significantly from chimpanzees (*p* = 0.0004) and mandrills (*p* = 0.0001), no significant differences were observed between the resistance profiles of the two non-human primate species (*p* = 0.0538). Unlike the host species, the distribution of resistance among phylogroups was not significant (global PERMANOVA *p* = 0.0913) (Figure 4).

## 3. Discussion

This study evaluated the potential impact of antibiotic use in NHPs and their close contact with personnel on the emergence and spread of AMR at the CIRMF Primate Center in Gabon. Most previous research on NHPs has focused on individuals who do not receive systematic antibiotic treatment, such as the chimpanzees studied in Tai, Côte d’Ivoire [16] and those in Kibale, Uganda [10]. The inclusion of two NHP groups (*Mandrillus sphinx* and *Pan troglodytes*) and the human staff within the same high-contact environment allowed us to capture this dynamic. The high resistance rates found in this study, especially when compared to data from wild or untreated with antibiotics NHP populations, underscore the potential role of institutional antibiotic use and frequent interspecies contact in driving these AMR levels. These findings are particularly concerning given the context of antibiotic use in human and veterinary medicine in Gabon, where agents such as tetracycline, ciprofloxacin, and trimethoprim are widely used and often easily accessible. Indeed, in this study, *Mandrillus sphinx* (n = 95) were significantly more representative than *Pan troglodytes* (n = 30). This sampling distribution accurately reflects the current composition of the primate colony housed at the CIRMF Primate Center, which comprises nearly 350 mandrills compared to around 40 chimpanzees [17].

The large population size and the semi-captive conditions of the mandrills, in particular, suggest a higher potential for human-animal contact and thus, increased risk of bidirectional bacterial exchange. *E. coli* is commonly used as an indicator organism for antimicrobial resistance because of its clinical importance and widespread occurrence. It readily circulates between humans, animals, and environmental compartments, enabling continuous exchange across these interconnected ecosystems. Due to its short generation time, *E. coli* plays an active role in horizontal gene transfer, acting as both a donor and a recipient of antimicrobial resistance determinants among microorganisms sharing the same ecological niche. Furthermore, its ability to acquire resistance genes strengthens its relevance in surveillance studies. Together, these characteristics justify the use of *E. coli* as a suitable model organism for the present study [3,4,5].

A high prevalence of *E. coli* isolates was observed, reaching 85.6% in NHPs and 83.3% in CDP staff. The isolation rate in NHPs observed in our study is slightly higher than the 85.3% previously reported in a similar context [18]. The observed prevalence of *E. coli* isolates in NHPs in our study is notably higher than the 70.7% reported by Clayton et al. (2014) [18] in primates at the Como Zoo. The high prevalence of *E. coli* in the non-human primates at the primatology center is not surprising, as the gastrointestinal tracts of NHPs are well-known to harbor *E. coli*, a ubiquitous commensal bacterium shared by both humans and animals.

The highest resistance in this study was observed for tetracycline, with an overall prevalence of 27.9% (34/122), followed by trimethoprim-sulfamethoxazoleat 20.5% (25/122). Importantly, the resistance rate for tetracycline was lower in NHPs than in humans, at 23.4% (25/107) and 60% (9/15), respectively. For instance, a 2021 study by Zhu et al. [19] on NHPs in zoos in China found higher frequencies for both, 62.7% resistance to tetracycline and 36.8% resistance to trimethoprim-sulfamethoxazole.

Furthermore, the prevalence of resistance to trimethoprim-sulfamethoxazole in our study is comparable to that found in the 2018 study by Weiss et al., conducted in humans and wild NHPs living near two national parks, with a prevalence of 20.3% [10]. The high resistance to these older, frequently used antibiotics in both the semi-captive NHPs and the staff strongly suggests selection pressure driven by local therapeutic practices.

The predominance of tetracycline resistance in both NHPs and human staff could be directly attributed to its widespread use in human and veterinary health within Gabon [20]. Similarly, the resistance observed to fluoroquinolones in NHPs may be influenced by the use of this family of antibiotics at the CDP.

It is plausible that the selective pressures exerted by these frequently administered antibiotics promote the emergence and propagation of resistant strains within the environment [21]. The overall prevalence of MDR *E. coli* isolates was 12.3% (15/122). The highest MDR burden was concentrated among CDP staff (40%, 6/15), followed by *Pan troglodytes* (15.4%, 4/26) and *Mandrillus sphinx* (6.2%, 5/81). The high MDR prevalence observed among staff is particularly concerning and may be partially attributable to inappropriate antibiotic use, particularly self-medication, increasing the risk of selecting resistant bacteria leading to the emergence of multi-resistant bacteria [22]. The nearly comparable resistance rates observed in chimpanzees and mandrills may, however, reflect the practical challenges noted by veterinarians in ensuring complete treatment compliance in these animals, particularly with oral follow-up regimens. Incomplete treatment is a well-recognized and major driver of antibiotic resistance development. These findings are concerning, as the prevalence of MDR isolates poses a significant public health threat by increasing the risk of therapeutic failure [23]. Furthermore, the rarity of *E. coli* isolates resistant to third- and fourth-generation cephalosporins and carbapenem is likely explained by the infrequent use of these agents by the veterinary staff at the CDP and the human staff.

This observation supports the hypothesis that the observed resistance profile is driven primarily by the selective pressure of antibiotics used locally and/or inappropriately.

This finding serves as a positive indicator of prudent antibiotic stewardship regarding this critical class of antimicrobials in the study area.

Phylo-group A was observed to be the most dominant group in both in NHPs isolates. Our results are consistent with those reported in a recent study conducted elsewhere in Gabon, which also showed that *E. coli* isolates from western lowland gorillas in Moukalaba-doudou Park belonged mainly to phylo-group A [12]. This dominance of commensal phylo-groups (A, B1, C, and D) in both semi-captive and wild Gabonese primates is expected, as these phylo-groups are commensal and commonly found in the gut microbiota of mammals. This finding is further relevant because phylo-group A was also the most common group found among the CDP staff.

However, the predominance of phylo-group A in the *E. coli* isolates from our NHPs, which are subjected to veterinary antibiotic administration, could support the hypothesis proposed by Mammeri et al. (2009) [24]. Their work suggested that the increased abundance of phylo-group A in domestic species may be due to their direct exposure to antibiotics, as it has been theorized that phylo-group A possesses a genetic heritage conducive to the acquisition and development of resistance genes. Given the high resistance rates and the dominance of phylo-group A in the CDP population, this association highlights a key mechanism by which AMR may be proliferating in this semi-anthropized environment.

Our results for the pathogenic phylo-groups differ significantly from those observed in NHPs in Gambia, where the majority of *E. coli* strains belonged to phylo-group B2, known to be associated with extraintestinal infections in humans and to carry factors linked to these diseases [11].

Finally, the shared presence of clade II in both humans and NHPs in our study could be considered indicative of environmental contamination, suggesting another pathway for bacterial exchange at the site [25]. Crucially, as was observed in our study, isolates carrying resistance were not limited to specific phylo-groups, but were rather distributed across all major phylo-groups [26]. The presence of such a wide range of phylo-groups in both NHPs and CDP staff poses a general threat to human and animal health. However, in NHPs, it often remains unclear whether certain phylo-groups function purely as commensals or possess pathogenic potential [18].

The varying prevalence of phylo-groups reported across different studies is highly context-dependent. These differences can be explained by numerous factors, including the host’s health status, dietary and genetic factors, environmental, social, and geographical conditions, or differences between sampling areas [27]. This reinforces the importance of context-specific studies, such as ours at the CDP, for understanding AMR dynamics.

This study clearly highlights the overlap of antibiotic-resistant *E. coli* and phylo-groups among NHPs and human staff at the CDP, a semi-anthropized setting that facilitates interaction.

Although the human sample size is limited, the similarity of resistance profiles between NHPs and staff, with nearly comparable resistance rates in *Mandrillus sphinx* (50.6%), *Pan troglodytes* (57.7%), and humans (80%), with *p* = 0.1064, strongly suggests the interspecies exchange of antibiotic-resistant *E*. *coli.* Tetracycline and trimethoprim-sulfamethoxazole were the antibiotics most affected by resistance. These results are consistent with findings from NHPs in captive settings, such as zoos in China [19], and rural areas in Uganda, where humans, domestic animals, and wildlife coexist [10]. The shared dominance of phylo-group A across all three populations further supports the existence of a common bacterial reservoir driven by anthropogenic activities and selective pressure from local antibiotic use at the center.

The application of multivariate analysis (ACP) provides a better understanding of the dynamics of AMR at the CIRMF Primate Centre. First, humans have the most diverse and intense resistance profiles, which probably reflects longer exposure to antibiotics in the community environment compared to the more controlled environment of non-human primates. Indeed, even though non-human primates are in close contact with humans, they retain a statistically distinct and more extensive resistance profile. It should be noted, however, that the difference is half as pronounced in chimpanzees as in mandrills, suggesting that the biological or behavioural proximity of chimpanzees may facilitate a convergence of their profiles towards those of humans. Nevertheless, the absence of a statistical difference between chimpanzees and mandrills (*p* = 0.0538) is a significant finding. It indicates a homogenization of resistance profiles among NHPs in captivity or semi-captivity, probably linked to the sharing of water resources or contact with staff.

The strong correlation between TET and SXT is particularly revealing, especially for the resistance observed to trimethoprim-sulfamethoxazole, which is not an antibiotic used for therapeutic purposes in non-human primates at the CDP. This strongly suggests either co-selection or direct transmission of resistant strains from humans to animals. On the other hand, the absence of significant clustering by phylo-groups suggests that AMR is not specific to a lineage at the human-primate interface. The wide dispersion of phylo-group A isolates across the resistance spectrum indicates that this dominant commensal phylo-group could act as a reservoir, capable of acquiring and maintaining various resistance genes.

Furthermore, the identification of a single isolate producing extended-spectrum beta-lactamase (ESBL) in *Mandrillus sphinx* is particularly concerning, especially since third- and fourth-generation cephalosporins are not used in non-human primates at the CDP. This finding reinforces the idea of transmission of resistant isolates from humans to animals, particularly when resistance appears in animals not exposed to these specific classes of antibiotics. This evidence reinforces the ‘One Health’ perspective, highlighting the deep interconnectedness of ecosystems between species. These findings underscore the critical importance of implementing comprehensive AMR surveillance programmes that simultaneously monitor all three compartments: humans, animals, and their shared environment.

## 4. Materials and Methods

### 4.1. Study Design, Area, and Population

This cross-sectional study was conducted between June and October 2023 at the Primatology Centre (CDP) of the CIRMF in southeastern Gabon (approx. 1°37′ N, 13°34′ E). For this cross-sectional study, the sample size was determined using the Cochran formula for finite populations to ensure statistical representativeness. Our samples are representative of our source populations, with 95 out of 350 mandrills, 30 out of 40 chimpanzees, and 18 out of 30 humans.

The study focused on three populations: the Central Africa chimpanzee subspecies (*Pan troglodytes troglodytes*) maintained under strict captive conditions, mandrills species (*Mandrillus sphinx*) maintained under semi-captive conditions, and the human staff at the CDP. The human study population was purposefully selected based on occupational proximity to non-human primates (NHPs). This cohort comprises animal caretakers and healthcare personnel, including veterinarians, engineers, and technicians. While caretakers maintain close daily contact through feeding and enclosure maintenance, healthcare personnel are involved in clinical procedures and biological sampling. These activities involve direct physical contact and potential exposure to bodily fluids, positioning this group as a critical high-risk interface for interspecies transmission.

### 4.2. Sample Collection and Data

**Figure 5:**
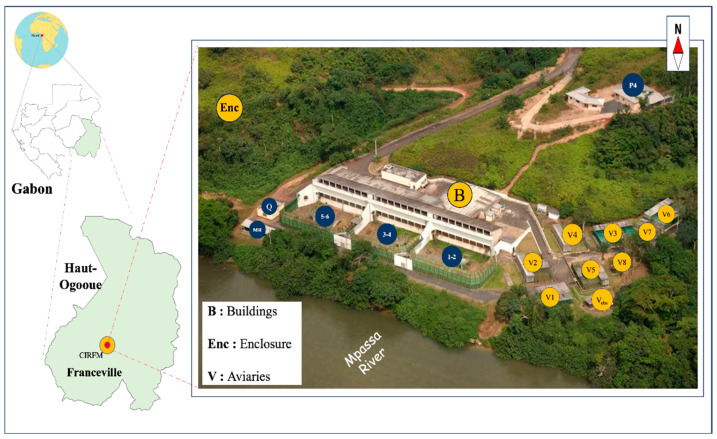
View of the site at CIRMF Primatology Centre (CDP). view of the site at CIRMF Primatology Centre (CDP). This figure has been adapted and modified from ref. [28] with the author’s permission. Legend: B 1–6: Buildings, Enc: enclosure, V1–8 and V_obs_: aviaries, Q: Quarantine, P4: Laboratory, and MR: Retirement home Aerial.

Fresh fecal samples from NHPs were collected non-invasively under veterinary supervision. The collection process involved observing the primates in their feeding areas for individual identification. Fecal samples were collected from various sites within the CDP, specifically the animal enclosure (Enc) and building (designated B, V1 to V8, B1 to B6) (Figure 5). Feces were collected using a sterile spatula, taking care to avoid any part that had been in contact with the ground to prevent environmental contamination. The spatula containing the sample was placed in the stool culture jar and then sealed tightly. For human staff participants, informed consent was obtained, and anonymized stool samples were collected in sterile stool culture jars. All fecal samples were collected in sterile containers and immediately transferred to a cooler (4 °C to 8 °C) to maintain microbial stability. Samples were transported to the laboratory and processed immediately to ensure strain viability and avoid the need for fecal freezing. Habitat and sex data of NHPs were collected in the field, while additional data concerning age, affiliation, origin, health records, and diet were obtained from a database managed by the veterinary staff.

### 4.3. Isolation and Identification of Escherichia coli Isolates by MALDI-TOF Mass Spectrometry

No pre-enrichment was performed. Each fresh stool sample was streaked onto Eosin Methylene Blue (EMB) agar (BioMérieux, Marcy l’Etoile, France) using aseptic technique in the CIRMF bacteriology laboratory. The agar plates were subsequently incubated at 37 °C for 24 h under aerobic conditions. Presumptive identification of *E. coli* was performed after incubation based on characteristic colony morphology (size, texture, blue-black color, and the presence of a green metallic sheen on EMB). Presumptive *E. coli* were then purified by culture on Trypcase Soy agar (TSA) at 37 °C for 24 h under aerobic conditions [29]. For long-term storage pending subsequent analysis, the suspected *E. coli* isolates were suspended in a Brain Heart Infusion—BHI (70%)/glycerol (30%) and stored at −20 °C.

The isolates were identified using matrix-assisted laser desorption ionization-time of flight (MALDI-TOF) mass spectrometry using the MALDI Biotyper database DB 8326 MSP (Bruker Daltonics, Bremen, Germany) in the Microbiology Laboratory of the Arnaud de Villeneuve Hospital in Montpellier (France) [30]. At this stage, a positive control was systematically used, the Bacterial Test Standard (BTS) (Ref: 8290190, Bruker). This standard, derived from an *E. coli* strain, was used to calibrate.

### 4.4. Antibiotic Susceptibility Testing

Antibiotic susceptibility was assessed using the Kirby–Bauer disk diffusion method on Müller–Hinton agar. The reading and interpretation of inhibition zone diameters were performed according to the recommendations of the European Committee on Antimicrobial Susceptibility Testing (EUCAST) [31]. Inocula corresponding to 0.5 McFarland were prepared using the Inoclic system (i2a, Montpellier, France). The bacterial suspension was then streaked onto a square Mueller-Hinton agar plate, and 30 antibiotic discs were placed on the agar using two antibiotic dispensers. The Mueller-Hinton agar plates containing the antibiotic discs were incubated in an oven for 18–24 h and placed in the ORION SIRscan automated system (i2a, Montpellier, France) [31]. The selection of antimicrobial agents was strategically determined to balance international standards with the clinical realities of Gabon. A total of 30 antibiotics were tested on each isolate, including penicillins: Amoxicillin (20 μg), ticarcillin (75 μg), piperacillin (30 μg), and temocillin (30 μg); penicillins with β-lactamase inhibitors: amoxicillin/clavulanic acid (20/10 μg), ticarcillin/clavulanic acid (75/10 μg), and piperacillin/tazobactam (30/6 μg); cephamycin: cefoxitin (30 μg); extended-spectrum cephalosporins: cefotaxime (5 μg), ceftazidime (10 μg), cefepime (30 μg), and cefpodoxime (10 μg); extended-spectrum cephalosporins with β-lactamase inhibitors: ceftazidime-avibactam (14 μg); carbapenems: imipenem (10 μg), ertapenem (10 μg), and meropenem (10 μg); monobactams: aztreonam (30 μg); quinolones: ofloxacin (5 μg), ciprofloxacin (5 μg), pefloxacin (5 μg), and levofloxacin (5 μg); aminoglycosides: gentamicin (10 μg), netilmicin (10 μg), tobramycin (10 μg) and amikacin (30 μg); folate pathway inhibitors: trimethoprim-sulfamethoxazole (1.25/23.75 μg); phenicol: fosfomycin (200 μg); phosphonic acids: chloramphenicol (30 μg); tetracyclines: tetracycline (30 μg); and polymyxins: colistin (50 μg). Extended-spectrum β-lactamase production was detected by using the combined double-disk synergy method [32]. Multidrug resistance (MDR) was defined according to international standards as non-susceptibility to at least one class in three or more antimicrobial categories [23].

#### Survey on Antibiotics Used in Human and Veterinary Health in Gabon

We conducted a targeted survey of medical and veterinary practitioners at the CIRMF to identify the specific molecules routinely administered to both human and primate populations in the region. By aligning our panel with local therapeutic practices, we aimed to correlate observed resistance profiles and the selective pressure of antibiotics. This approach provided a critical baseline for distinguishing between anthropogenic (acquired) resistance resulting from direct antibiotic use and the natural background of environmental resistance. The antimicrobial panel was informed by pharmaceutical records provided by the facility’s veterinary and medical staff. In the NHP cohort, documented antibiotic use included first-generation cephalosporins (cephalexin), natural and penicillinase-resistant penicillins (penicillin and oxacillin), fluoroquinolones (enrofloxacin), macrolides (erythromycin), lincosamides (lincomycin), and phosphonic acids (chloramphenicol). Simultaneously, the human medical staff identified several key antibiotics routinely prescribed to personnel, including beta-lactamase inhibitor combinations (amoxicil-lin-clavulanic acid), penicillinase-resistant penicillins (flucloxacillin), third-generation cephalosporins (cefixime), sulfonamides (trimethoprim-sulfamethoxazole), fluoroquinolones (ofloxacin), and macrolides (azithromycin). Antibiotics used in human and animal health are penicillins (amoxillin), fluoroquinolones (ciprofloxacin) and tetracyclines (tetracycline and doxycycline).

### 4.5. Molecular Analysis

#### DNA Extraction and Phylo-Groups Characterizations

Genomic DNA was extracted using the boiling method [33]. *E. coli* phylo-groups were characterized using the Clermont quadruplex PCR method, targeting the *chuA, yjaA*, TspE4.C2, and *arpA* genes [34] (Table 1). This allows for the assignment of *E. coli* isolates into eight distinct phylo-groups: A, B1, B2, C, D, E, F, and clade I. The *arpA* and *trpA* genes were subjected to simplex PCR to confirm phylo-groups E and C, respectively. Amplifications were performed using a Thermal cycler (Eppendorf AG 22331 Hamburg, Germany) under the following conditions: initial denaturation at 94 °C for 4 min, 30 cycles (denaturation at 94 °C for 30 s, hybridization at 57 °C for group E and 59 °C for group C and quadruplexes for 30 s, elongation at 72 °C for 5 min). The final elongation step was performed at 72 °C for 7 min. The PCR products were separated on a 2% agarose gel containing SYBR^®^ Safe DNA dye (Invitrogen, Thermo Fisher Scientific, Waltham, MA, USA) with a molecular weight marker (100 bp DNA Ladder, New England Biolabs, Ipswich, MA, USA). The products amplified by multiplex PCR to characterize the phylo-groups of *E. coli* are shown in Figure S1, and the assignment of *E. coli* isolates to different phylo-groups was performed using a quadruplex phylo-typing method (Table S1). While a reference strain was not included in every PCR run, the validity of the results was maintained through several rigorous quality control measures. First, the systematic inclusion of negative controls (nuclease-free water) effectively ruled out reagent or laboratory contamination. Second, the specificity of all amplifications was verified by comparing the obtained amplicon sizes against expected theoretical molecular weights, visualized using a high-resolution molecular weight ladder. The high degree of concordance between observed and predicted band sizes ensures the fidelity of the gene detection.

### 4.6. Statistical Tests

All collected data were stored and managed using an Excel (version16.0) database. Statistical analyses and visualizations were performed using R (version 4.3.1) and Python (version 3.9.23). The chi-square test was used to compare qualitative data (resistance status and *E. coli* phylogroups) between the three populations studied (CDP staff, *Pan troglodytes* and *Mandrillus sphinx*). Differences were considered statistically significant for a *p*-value less than 0.05. Graphical representations were generated using matplotlib (v3.9.4) and seaborn (v0.13.2).

## 5. Conclusions

The similarity observed in antibiotic resistance profiles of *E. coli* in *Mandrillus sphinx*, *Pan troglodytes*, and CDP staff strongly suggests the likelihood of bidirectional transmission of antibiotic-resistant isolates, facilitated by the high-contact environment and promiscuity at the CDP. The documented presence of MDR isolates and a critical ESBL-producing isolate highlights the significant risk of resistant bacteria dissemination between species in this setting. These crucial data reinforce the importance of the One Health approach. They will be paramount in enabling the development of targeted strategies to combat AMR at CDP, thereby serving the dual objective of protecting human public health and conserving non-human primate populations.

Limitations: Despite the importance of our findings, this study has certain limitations, namely the small sample size of humans. To overcome this, future studies involving a larger human sample, including surrounding communities, would allow these conclusions to be broadened.

## Figures and Tables

**Table 1:** Primer sequences and sizes of PCR products used in the phylo-typing method.

PCR Reaction	Primer ID	Target	Primer Sequences (5′→ 3′)	bp
Quadruplex	chuA. 1b	*chuA*	ATGGTACCGGACGAACCAAC	288
	chuA.2		TGCCGCCAGTACCAAAGACA	
	yjaA. 1b	*yjaA*	CAAACGTGAAGTGTCAGGAG	211
	yjaA. 2b		AATGCGTTCCTCAACCTGTG	
	TspE4C2.1b	TspE4C2	CACTATTCGTAAGGTCATCC	152
	TspE4C2.2b		AGTTTATCGCTGCGGGTCGC	
	AceK.F	*arpA*	AACGCTATTCGCCAGCTTGC	400
	ArpA1.R		TCTCCCCATACCGTACGCTA	
Group E	ArpAgpE.f	*arpA*	GATTCCATCTTGTCAAAATATGCC	301
	ArpAgpE.r		GAAAAGAAAAAGAATTCCCAAGAG	
Group C	trpAgpC.1	*trpA*	AGTTTTATGCCCAGTGCGAG	219
	trpAgpC.2		TCTGCGCCGGTCACGCCC	

This table represents the different primers used to identify phylo-groups.

## Data Availability

The data are not publicly available due to ethical and privacy restrictions.

## Supplementary Materials

The following supporting information can be downloaded at: https://www.mdpi.com/article/10.3390/antibiotics15050446/s1, Figure S1: Multiplex PCR profiles for the phylogrouping of *E. coli* isolates; Table S1: Criteria for assigning *E. coli* phylogroups by quadruple multiplex PCR.
