# Supplementary material for: Antimicrobial Resistance and Phylo-Groups of Escherichia coli at the Human–Primate Interface in Gabon: A One Health Study
Source: Antibiotics (Basel). 2026 Apr 29;15(5):446. doi: 10.3390/antibiotics15050446 (PMC13203282; doi:10.3390/antibiotics15050446)
Supplement: Supplementary file 1 [file antibiotics-15-00446-s001.zip › antibiotics-4155739-supplementary.pdf]

Supplementary Materials

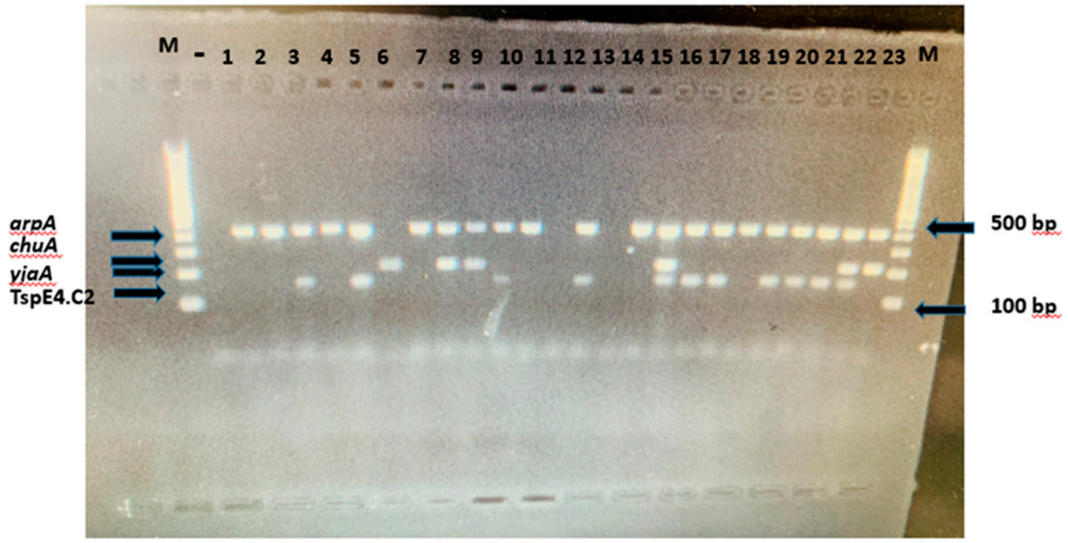

Figure S1: Multiplex PCR profiles for the phylogrouping of *E. coli* isolates.

The agarose gel (2%) shows the amplicons obtained for the arpA, chuA, yjaA and TspE4.C2 fragments. Lanes M: 100 bp molecular weight marker; - : negative control ; Lanes 2-23: E. coli isolates tested. PCR reactions were performed in a 20 ml volume containing 2X master mix (Invitrogen, Thermo Fisher Scientific, USA), 3 ml of DNA and the specific primers. The quantities of primers used were 20 pmol, with the exception of AceK.f (40 pmol), ArpA1.r (40 pmol), trpBA.f (12 pmol) and trpBA.r (12 pmol).

Table S1: Criteria for assigning *E.coli* phylogroups by quadruple multiplex PCR

Quadruplex genotype				
arpA (400 bp)	*chuA* (288 bp)	*yjaA* (211 bp)	TspE4.C2 (152 bp)	Phylo-group
+	−	−	−	A
+	−	−	+	B1
−	+	−	−	F
−	+	+	−	B2
−	+	+	+	B2
−	+	−	+	B2
+	−	+	−	A or C
+	+	−	−	D or E
+	+	−	+	D or E
+	+	+	−	E or clade I
−	−	+	−	Clade I or II
−	(476)[c]	−	−	Clade III, IV or V
−	−	−	+	Unknown
−	−	+	+	Unknown
+	−	+	+	Unknown
+	+	+	+	Unknown
−	−	−	−	Unknown

This table details the presence (+) or absence (−) profiles of the four target genetic markers *arpA* (400 bp), *chuA* (288 bp), *yjaA* (211 bp) and the TspE4.C2 DNA fragment (152 bp) used to identify the main phylogenetic lineages. The interpretation of band combinations allows isolates to be classified according to the Clermont method.
